# The oncogenic role of ecotropic viral integration site 1 in hematological malignancies: mechanisms of activation and leukemogenesis

**DOI:** 10.3389/fimmu.2026.1750231

**Published:** 2026-02-16

**Authors:** Yuchao Hao, Jing Liu, Jiacheng Lou, Jinsong Yan

**Affiliations:** 1 Department of Hematology, Liaoning Medical Center for Hematopoietic Stem Cell Transplantation, The Second Hospital of Dalian Medical University, Liaoning Key Laboratory of Hematopoietic Stem Cell Transplantation and Translational Medicine, Blood Stem Cell Transplantation Institute, Diamond Bay Institute of Hematology, The Second Hospital of Dalian Medical University, Dalian, China; 2 Department of Pediatrics, Second Hospital of Dalian Medical University, Dalian, China; 3 Department of Hematology, YingKou People’s Hospital, Yingkou, Liaoning, China

**Keywords:** 3q26 rearrangement, EVI1, hematopoiesis, leukemia, oncogene, poor prognosis, transcription factor

## Abstract

Ecotropic viral integration site 1 (EVI1), encoded by the EVI1 gene on chromosome 3q26.2, is a dual-domain zinc finger transcription factor that functions as a potent proto-oncogene in a wide spectrum of hematological malignancies. Under normal physiological conditions, its expression is tightly regulated and restricted primarily to hematopoietic stem cells and specific embryonic tissues. However, aberrant overexpression of EVI1 is a hallmark of aggressive myeloid leukemias, including acute myeloid leukemia (AML), myelodysplastic syndromes (MDS), and the blast crisis of chronic myeloid leukemia (CML). The oncogenic activation of EVI1 occurs through diverse genetic mechanisms, most notably chromosomal rearrangements involving the 3q26 locus, such as inv(3)(q21q26.2) and t(3;3)(q21;q26.2), which juxtapose the EVI1 gene with potent enhancers like that of GATA2. Other mechanisms include the formation of oncogenic fusion genes (e.g., AML1-EVI1, ETV6-EVI1), enhancer hijacking, and retroviral insertional mutagenesis. Once overexpressed, EVI1 drives leukemogenesis through multifaceted molecular actions. It acts as a master transcriptional regulator, profoundly disrupting normal hematopoietic differentiation by repressing key lineage-specific transcription factors like RUNX1 and interfering with cytokine-induced maturation. Concurrently, EVI1 promotes cell survival and proliferation by modulating critical signaling pathways, including the potent inhibition of the tumor-suppressive TGF-β pathway and the activation of the pro-survival PI3K/AKT/mTOR cascade via PTEN suppression. EVI1 also cooperates with a multitude of other oncogenic lesions, such as MLL rearrangements, AML1 mutations, and activated RAS signaling, to accelerate disease progression. Clinically, EVI1 overexpression is one of the most robust independent indicators of poor prognosis, associated with therapy resistance and reduced overall survival. This review provides a detailed discussion of the mechanisms underlying EVI1’s activation, its complex molecular functions in hematopoietic transformation, and its profound clinical implications in hematological malignancies.

## 1 Introduction

Hematological malignancies represent a diverse group of cancers affecting the blood, bone marrow, and lymphoid systems, characterized by the clonal expansion of hematopoietic cells arrested at various stages of differentiation. The molecular pathogenesis of these diseases is complex, involving a stepwise accumulation of genetic and epigenetic alterations that dysregulate fundamental cellular processes such as proliferation, survival, and differentiation. Among the myriads of oncogenes implicated in these disorders, Ecotropic viral integration site 1 (EVI1) has emerged as a particularly powerful and clinically significant driver of aggressive myeloid neoplasms.

The EVI1 gene, also known as PRDM3, is located on human chromosome 3q26.2. It was first identified as a common site of retroviral integration in murine myeloid leukemias, where viral promoter/enhancer insertion led to its transcriptional activation and subsequent malignant transformation (1–3). In human hematology, its notoriety stems from its strong association with chromosomal abnormalities involving the 3q26 band, which are linked to some of the most aggressive forms of acute myeloid leukemia (AML), myelodysplastic syndromes (MDS), and the progression of chronic myeloid leukemia (CML) to blast crisis (4).

EVI1 is a transcription factor characterized by two distinct zinc finger domains: a proximal domain with seven zinc fingers and a more distal domain with three. This structure allows it to bind DNA in a sequence-specific manner and regulate the expression of a vast network of target genes (5, 6). The EVI1 locus is complex, also encoding a longer fusion transcript, MDS1-EVI1, which includes an N-terminal PR/SET domain with potential histone methyltransferase activity (7, 8). The differential expression and function of these isoforms add layers of complexity to EVI1’s biological roles.

Functionally, EVI1 acts as a master regulator of hematopoietic stem and progenitor cells (HSPCs). Its overexpression blocks cellular differentiation in response to hematopoietic cytokines, promotes self-renewal, and confers a profound survival advantage, thereby setting the stage for leukemic transformation (9). This is achieved through its ability to reprogram the cellular transcriptome and interfere with critical tumor-suppressive signaling pathways. Clinically, the presence of EVI1 overexpression, regardless of the underlying genetic mechanism, consistently correlates with resistance to conventional chemotherapy and is recognized as one of the most adverse prognostic factors in AML (10–12).

This review will provide a detailed and comprehensive examination of the multifaceted role of EVI1 in hematological malignancies. We will first explore the diverse molecular mechanisms responsible for its oncogenic activation. Subsequently, we will delve into the intricate pathogenic functions of EVI1, detailing its impact on hematopoietic differentiation, signal transduction, epigenetic regulation, and its cooperation with other oncogenic drivers. Finally, we will discuss the profound clinical implications of EVI1 expression and its establishment as a critical biomarker and a high-priority therapeutic target.

## 2 The EVI1 gene and protein: structure and isoforms

**Figure 1:**
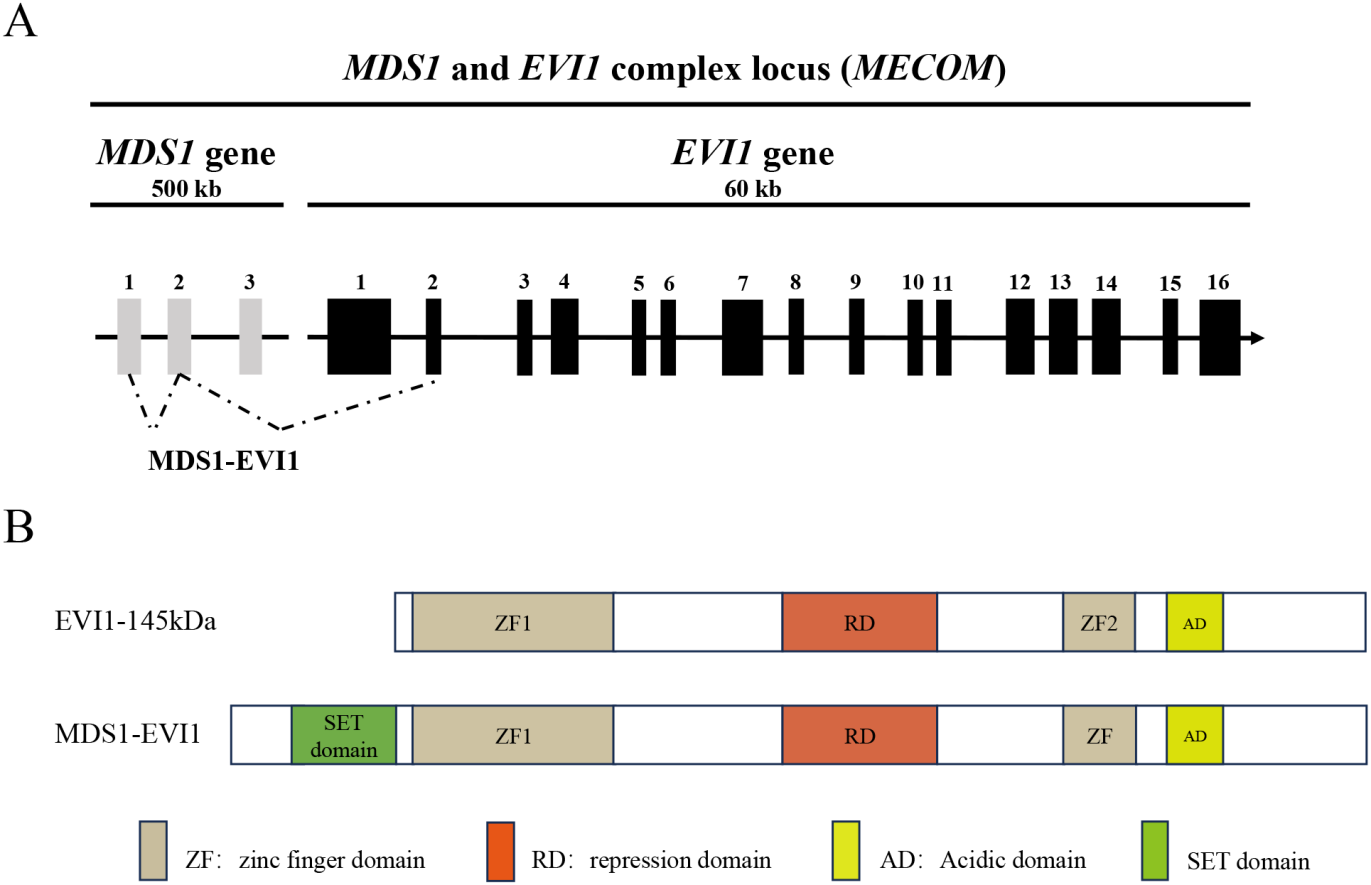
Structure of *EVI1* isoforms. **(A)** Schematic representation of the human *MECOM* locus. The alternative intergenic splicing between the second exon of MDS1 and the second exon of EVI1 is represented by dashed lines. The alternative intragenic splicing variants of EVI1 are represented by lines. **(B)** Structure of the EVI1 isoforms as a result of the alternative splicings. MDS1-EVI1 has an additional PR/SET domain. Adapted from (8).

The EVI1 gene locus at chromosome 3q26.2 is transcriptionally complex, giving rise to several protein isoforms with distinct, and sometimes opposing, functions (**Figure 1**). The two major protein products are EVI1 itself and a larger fusion protein known as MDS1-EVI1. The canonical EVI1 protein is a 145 kDa nuclear protein that functions primarily as a transcriptional repressor, although it can also act as an activator depending on the cellular context and promoter architecture (8). Its structure is defined by two separated zinc finger domains. The N-terminal domain contains seven C2H2-type zinc fingers (ZF1) that mediate binding to specific DNA consensus sequences, while the C-terminal domain contains three zinc fingers (ZF2) (13). Separating these two DNA-binding domains is a repression domain, and a central acidic domain is also present. This dual-domain structure allows EVI1 to engage in complex protein-protein and protein-DNA interactions, enabling it to function as a scaffold for larger regulatory complexes (14).

In contrast, the MDS1-EVI1 transcript arises from the splicing of exons from the upstream MDS1 gene to the second exon of EVI1. This results in a larger protein that shares the entire EVI1 sequence but contains an additional 188 amino acids at its N-terminus. This N-terminal extension includes a PR/SET (PRDI-BF1 and RIZ) domain, which is homologous to domains found in histone methyltransferases (15). The presence of this PR domain suggests a role in epigenetic regulation through chromatin modification. Functionally, MDS1-EVI1 has been shown to act as a transcriptional activator and can have distinct biological effects compared to the shorter EVI1 isoform (16). For instance, while EVI1 is a potent inhibitor of the TGF-β signaling pathway, MDS1-EVI1 has been reported to enhance TGF-β signaling, highlighting the functional divergence of these isoforms (17). Another isoform, MEL1 (MDS1/EVI1-like gene 1), also known as PRDM16, is a close homolog of MDS1/EVI1 and is similarly activated in certain leukemias (18). A shorter isoform of MEL1, MEL1S, lacks the PR domain and has been shown to potently block G-CSF-induced myeloid differentiation, a function reminiscent of EVI1 (19). The balance between the expression of these different isoforms can therefore significantly influence the cellular phenotype and the course of disease.

## 3 Mechanisms of EVI1 oncogenic activation

In normal hematopoiesis, EVI1 expression is very low or undetectable in mature blood cells. Its oncogenic potential is unleashed through aberrant overexpression, which is driven by a variety of distinct genomic events. These events disrupt the gene’s normal regulatory landscape, leading to its constitutive high-level expression in hematopoietic progenitor cells.

### 3.1 Chromosomal rearrangements of the 3q26 locus

The most well-characterized mechanism of EVI1 activation is through chromosomal rearrangements involving its locus at 3q26.2 (**Table 1**). The classic rearrangements are the pericentric inversion inv(3)(q21q26.2) and the balanced translocation t(3;3)(q21;q26.2). These abnormalities are found in approximately 2-3% of AML cases and are strongly associated with a unique clinical phenotype, often including normal or elevated platelet counts and multilineage dysplasia, particularly of the megakaryocytic lineage (20). Both inv(3) and t(3;3) are classified as high-risk aberrations conferring an extremely poor prognosis (20, 21).

**Table 1:** Summary of the EVI1 activation mechanism mediated by 3q26 rearrangement.

Description	Content/Function	Reference
MECOM (EVI1)	The high expression of key oncogenes is an independent adverse prognostic factor in AML/MDS. As a transcription factor, it reshapes the transcriptional program by recruiting co-inhibitory factors (such as CTBP), inhibits differentiation and enhances self-renewal	(22–24)
3q26 rearrangement (inv(3), t(3;3))	The most classic activation mechanism of MECOM leads to enhancer hijacking. It is the independent subtype classified by WHO and associated with myeloid malignancy and TKI resistance in CML	(20, 25, 26)
MYB	The transcription factor specifically binds to a key regulatory element of approximately 700bp within the hijacked G2DHE. Its combination is crucial for driving EVI1 expression and is a potential specific therapeutic target.	(27)
CTBP1/CTBP2	The key co-inhibitory factor of EVI1. EVI1 binds to the CTBP protein through its PLDLS motif, and this interaction is indispensable for the transformation and maintenance of leukemia.	(23)
Therapeutic targets	1. BET bromine domain inhibitors (such as JQ1) target the super enhancer properties of G2DHE. 2. MYB inhibitors, blocking the interaction between MYB and co-activators, specifically down-regulate EVI1.	(27, 28)

**Figure 2:**
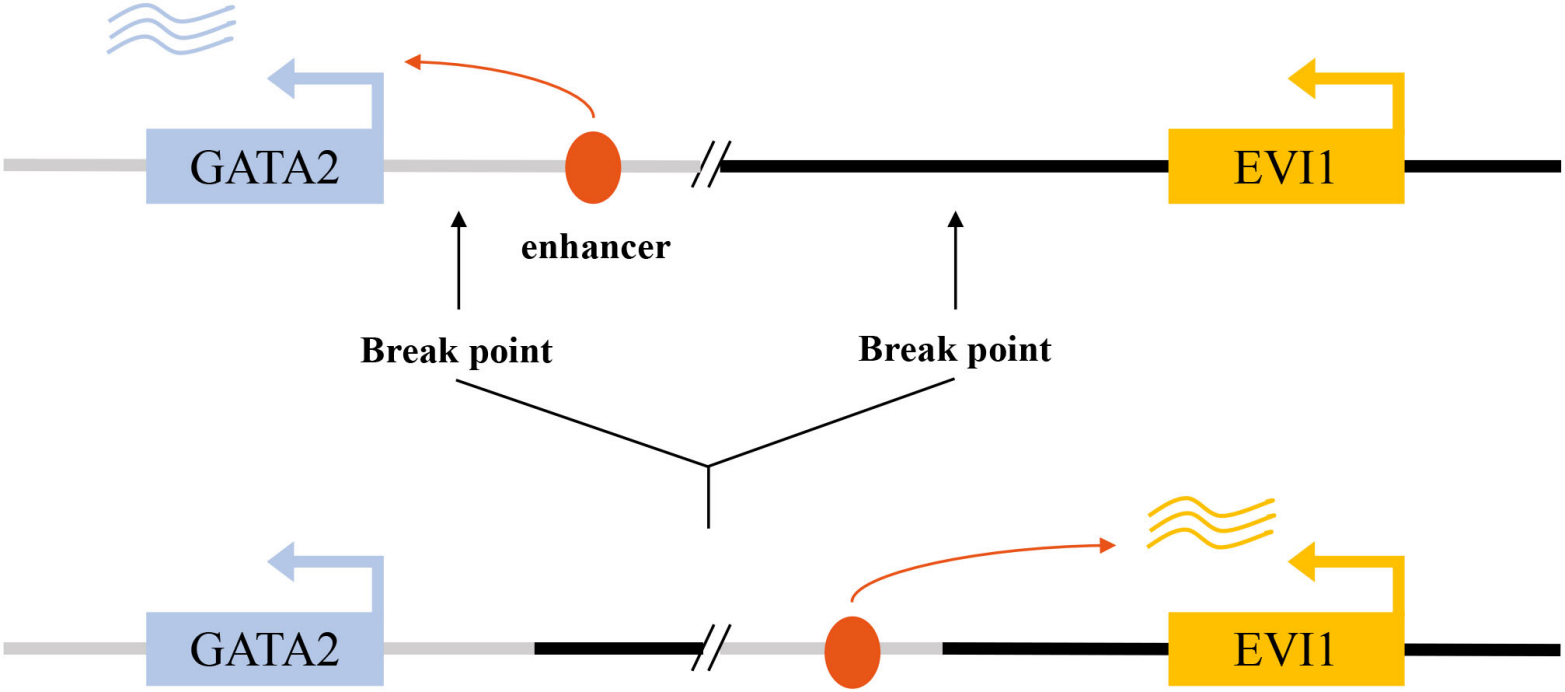
Enhancer hijacking drives aberrant EVI1 activation in inv(3)/t(3;3) acute myeloid leukemia. In the normal genomic context, a GATA2-associated enhancer at the 3q21 locus regulates physiological GATA2 transcription (upper panel). In inv(3)(q21q26) or t(3;3)(q21;q26) AML, chromosomal rearrangements relocate this enhancer to the MECOM/EVI1 locus, resulting in aberrant enhancer–promoter interaction and constitutive EVI1 expression (lower panel).

The primary molecular consequence of these rearrangements is the repositioning of a distal hematopoietic enhancer of the GATA2 gene, located at 3q21, into close proximity with the EVI1 promoter at 3q26.2 (29). This GATA2 enhancer is highly active in hematopoietic stem and progenitor cells, and its hijacking by the EVI1 locus drives massive, lineage-inappropriate overexpression of EVI1 (2, 30). This mechanism exemplifies a phenomenon known as “enhancer hijacking,” where a potent regulatory element is aberrantly co-opted to drive the expression of a proto-oncogene (**Figure 2**). In addition to these classic 3q abnormalities, other, more cryptic or atypical rearrangements involving the 3q26 band have been identified that similarly result in EVI1 overexpression and are associated with a clinical course resembling that of classic inv(3) AML (31). The consistent outcome of these diverse rearrangements is the deregulation of EVI1, establishing it as the critical pathogenic driver associated with 3q26 abnormalities. This mechanism also often leads to haploinsufficiency of GATA2, further contributing to the hematopoietic defects observed in these patients.

### 3.2 Fusion gene formation

A second major mechanism for EVI1 activation is the formation of chimeric fusion genes through chromosomal translocations that fuse the coding sequence of EVI1 with that of another gene. Besides the classical inv(3)/t(3;3), a number of other 3q26 rearrangements with poor treatment response have been reported in AML (31). These fusions typically place the EVI1 gene under the control of the fusion partner’s promoter, leading to its ectopic expression, and create a novel protein with combined or altered functions (**Table 2**).

**Table 2:** Fusion gene involving MECOM (EVI1) in acute myeloid leukemia.

Description	Partner gene/element	Molecular consequence/function	Frequency in AML	Reference
t(3;21)(q26;q22)	RUNX1 (AML1)	RUNX1::MECOM fusion disrupts RUNX1-dependent differentiation programs; plays a significant role in leukemogenesis	Rare; enriched in therapy-related AML	(32–34)
t(3;12)(q26;p13)	ETV6 (TEL)	Formation of ETV6::MECOM fusion; altered transcriptional repression and impaired hematopoietic differentiation	Rare (<0.5%)	(35)
t(3;8)(q26;q24)	MYC	EVI1 overexpression is a result of ‘enhancer hijacking’ of the MYC superenhancer, which is facilitated by CTCF-mediated loops	Rare	(36)
t(2;3)(p21;q26)	THADA	EVI1 overexpression without clear fusion partner; likely regulatory rearrangement	Very rare	(37)

One of the most studied fusions is the t(3;21)(q26;q22), which generates the AML1-EVI1 (also known as RUNX1-EVI1) fusion gene. This fusion is recurrently found in therapy-related MDS/AML and CML in blast crisis. The resulting AML1-EVI1 chimeric protein contains the N-terminal portion of the transcription factor AML1 (including its DNA-binding Runt domain) fused to the majority of the EVI1 protein (33, 38). This fusion protein has a dual, devastating impact. It acts as a dominant-negative inhibitor of wild-type AML1, a master regulator of normal hematopoiesis, thereby blocking differentiation (39). Simultaneously, it brings the potent oncogenic functions of EVI1 into the cell. The AML1-EVI1 fusion protein has been shown to efficiently transform hematopoietic stem cells and cause embryonic hematopoietic abnormalities in mouse models, underscoring its potent leukemogenic capacity (33, 40). The fusion protein also aberrantly sequesters the AML1 binding partner PEBP2β in the nucleus, further disrupting normal hematopoietic regulation (41).

Other translocations also create pathogenic EVI1 fusions. The t(3;12)(q26;p13) results in an ETV6-EVI1 (also known as TEL-EVI1) fusion, which is associated with CML and other myeloproliferative disorders with poor prognosis (42). The ETV6 component provides a potent promoter driving EVI1 expression and a self-association domain that may enhance the oncogenic activity of the fusion protein (43). Similarly, the t(2;3)(p15-22;q26) and t(3;6) can lead to EVI1 overexpression or fusion events associated with poor-risk AML (37). A fusion between RPN1 and EVI1 has also been described in patients with inv(3) or t(3;3), contributing to the adverse prognosis (21).

### 3.3 Enhancer hijacking and other regulatory mechanisms

Beyond the classic GATA2 enhancer hijacking, EVI1 can be activated by co-opting other powerful regulatory elements. A compelling example is the hijacking of a MYC super-enhancer by the EVI1 locus in a subset of AML cases. This event leads to the formation of a neotad (a newly formed topologically associating domain) that places EVI1 under the control of a potent enhancer normally reserved for the MYC oncogene, resulting in massive EVI1 overexpression and aggressive leukemia (36). In T-cell acute lymphoblastic leukemia (T-ALL), translocations involving the T-cell receptor beta (TCRβ) locus can bring its powerful enhancer to the vicinity of EVI1, driving its expression and causing undifferentiated leukemia (44). These examples illustrate a common theme in EVI1 deregulation: its promoter is relatively weak and requires the illicit appropriation of strong, lineage-specific enhancers to achieve oncogenic levels of expression. Furthermore, comprehensive genomic analyses using RNA sequencing are continually improving the detection of both canonical and cryptic EVI1 rearrangements and fusion events in AML, providing a more complete picture of its activation landscape (45).

### 3.4 Retroviral insertional mutagenesis

The historical discovery of EVI1 as an oncogene came from studies of murine leukemia models, where retroviral integration into the host genome served as a powerful tool for identifying cancer-causing genes. Insertions of retroviral long terminal repeats (LTRs), which contain strong promoter and enhancer elements, in or near the Evi1 gene locus were found to be a common event in myeloid leukemias induced by these viruses (3). This insertional mutagenesis provided the first direct evidence that aberrant activation of EVI1 expression is a primary oncogenic event. Similar insertional activation of the EVI1 homolog Prdm16 has also been shown to induce leukemia (46). These preclinical models have been instrumental in establishing the cause-and-effect relationship between EVI1 overexpression and leukemogenesis and continue to be valuable tools for studying its function (47).

## 4 Molecular pathogenesis driven by EVI1 overexpression

**Figure 3:**
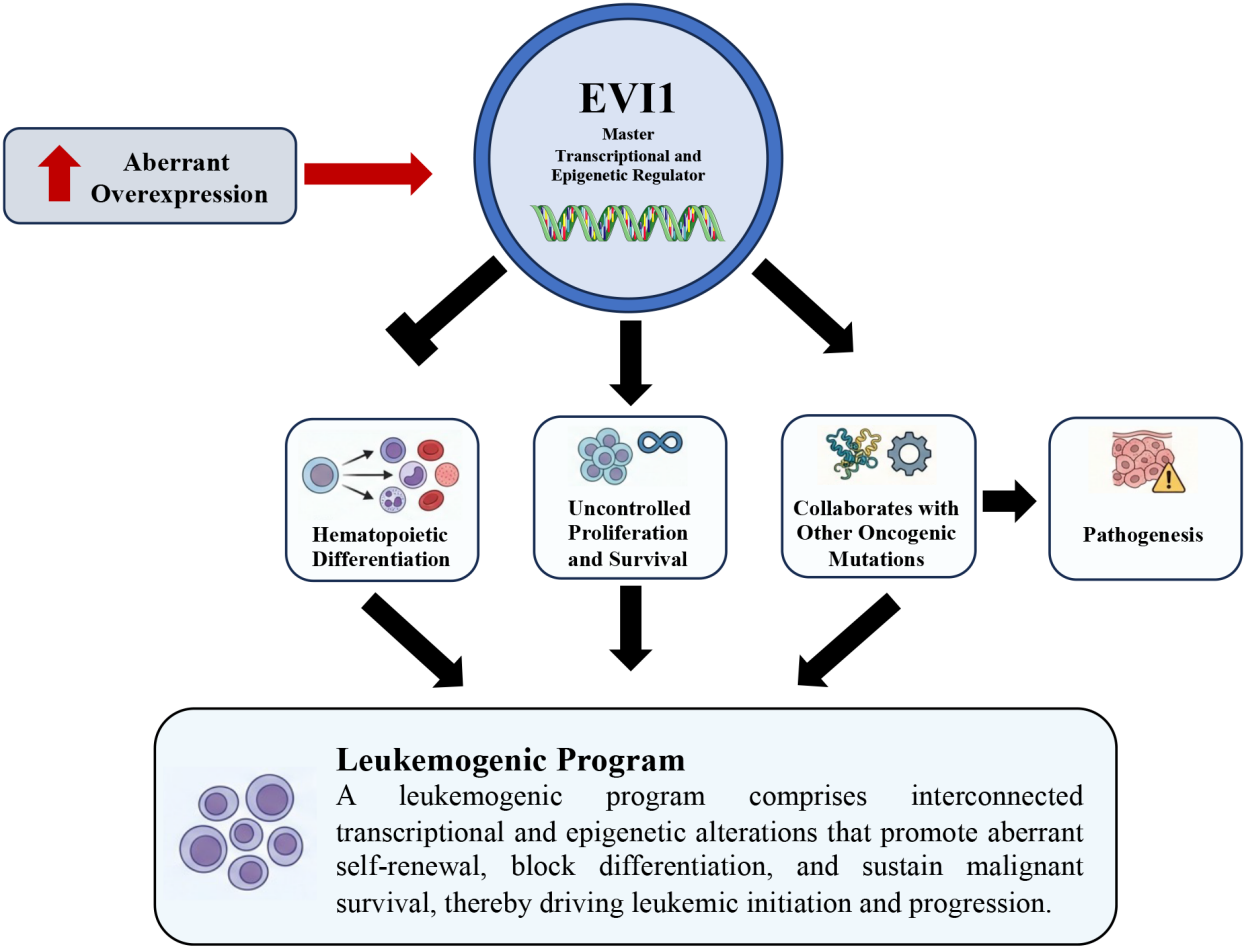
EVI1-driven leukemogenic program in acute myeloid leukemia. Aberrant overexpression of EVI1 functions as a master transcriptional and epigenetic regulator that initiates and sustains a leukemogenic program. Elevated EVI1 activity disrupts normal hematopoietic differentiation, promotes uncontrolled proliferation and survival, and cooperates with additional oncogenic alterations to drive leukemic pathogenesis. Collectively, these interconnected transcriptional and epigenetic events establish a leukemogenic program that underlies leukemia initiation and progression.

Once aberrantly overexpressed, EVI1 orchestrates a comprehensive leukemogenic program by functioning as a master transcriptional and epigenetic regulator. It drives pathogenesis by blocking hematopoietic differentiation, promoting uncontrolled proliferation and survival, and collaborating with other oncogenic mutations (**Figure 3**).

### 4.1 Disruption of hematopoietic differentiation and proliferation

A cardinal feature of EVI1-driven leukemia is a profound block in myeloid differentiation. EVI1 exerts this effect by repressing the expression and/or function of key transcription factors that are essential for normal hematopoiesis. One of its most critical targets is RUNX1 (AML1), a master regulator of definitive hematopoiesis. EVI1 directly binds to and represses the RUNX1 promoter and also physically interacts with the RUNX1 protein to inhibit its transcriptional activity, effectively shutting down its lineage commitment programs (48). This antagonism is a central node in EVI1-mediated leukemogenesis. Similarly, EVI1 interferes with differentiation induced by hematopoietic cytokines like granulocyte colony-stimulating factor (G-CSF) (19).

EVI1 also skews hematopoietic output. Its forced expression in hematopoietic progenitors can induce abnormal megakaryocytic differentiation, consistent with the thrombocytosis often seen in patients with 3q abnormalities (49). This is partly mediated by its influence on the TPO/MPL signaling pathway, which promotes the growth of EVI1-positive, CD41-positive megakaryocytic cells (50). At the stem cell level, EVI1 helps maintain the self-renewal and undifferentiated state of hematopoietic stem cells (HSCs), in part by upregulating the adhesion G-protein coupled receptor GPR56, which is critical for HSC maintenance (51). It also regulates the expression of other key hematopoietic regulators, including GATA-1, GATA-2, and SCL/TAL1 (52), and the transcription factor PLZF (53), further cementing its role as a global disruptor of the normal hematopoietic hierarchy. By blocking differentiation while promoting self-renewal, EVI1 effectively traps hematopoietic cells in an immature, proliferative state, a crucial step toward malignant transformation (9).

### 4.2 Interference with key signaling pathways

In addition to disrupting transcriptional networks governing differentiation, EVI1 promotes cell survival and proliferation by rewiring intracellular signaling pathways.

A primary mechanism is the potent inhibition of the Transforming Growth Factor-beta (TGF-β) signaling pathway, a critical tumor-suppressive pathway that normally induces cell cycle arrest and apoptosis. EVI1 accomplishes this through multiple interactions. It physically binds to Smad3, a key signal transducer of the TGF-β pathway, preventing it from participating in active transcriptional complexes (54). Furthermore, EVI1 recruits the transcriptional co-repressor C-terminal Binding Protein (CtBP) to the promoters of TGF-β target genes, leading to their active repression (55). The post-translational phosphorylation of EVI1 has been shown to enhance its interaction with CtBP, thereby strengthening its repressive function (56). By dismantling this crucial tumor suppressor checkpoint, EVI1 provides cells with a powerful survival and proliferative advantage. Interestingly, the AML1-EVI1 fusion protein also effectively blocks TGF-β signaling, contributing to its oncogenic activity (17).

EVI1 also promotes cell survival by activating the PI3K/AKT/mTOR pathway. It achieves this by transcriptionally repressing the tumor suppressor gene PTEN. The loss of PTEN function leads to the accumulation of PIP3 and subsequent constitutive activation of AKT and its downstream effector mTOR, a central regulator of cell growth, proliferation, and survival (57). More recently, EVI1 was shown to drive mTORC1 activation through a novel axis involving the upregulation of the histone demethylase Kdm6b, which in turn activates the expression of Laptm4b, a lysosomal protein that promotes mTORC1 signaling (58). This sustained activation of pro-survival signaling pathways makes EVI1-expressing cells highly resistant to apoptosis.

### 4.3 Transcriptional and epigenetic reprogramming

As a DNA-binding protein, EVI1’s core function is to reprogram the cellular transcriptome and epigenome. It acts as a scaffold for large protein complexes that modify chromatin and regulate gene expression (**Table 3**). EVI1 interacts with histone methyltransferases such as SUV39H1 and G9a, recruiting them to target gene promoters to mediate transcriptional repression through the deposition of repressive histone marks (e.g., H3K9 methylation) (59). This function is critical for its ability to immortalize bone marrow cells.

**Table 3:** Summary of downstream targets and molecular mechanisms of EVI1.

Description	Function	Reference
CEBPA	inhibits CEBPA transcription by binding to its enhancer, thereby blocking granulocyte differentiation	(7)
RUNX1	1) Interact directly with the RUNX1 protein 2) Bind to RUNX1 promoter to inhibit its transcription	(48, 60)
GATA-1	MECOM/EVI1 inhibits GATA-1-mediated transcriptional activation through physical interaction, thereby blocking erythroid differentiation	(61, 62)
GATA2	In inv(3) AML, the ectopic GATA2 enhancer drives high expression of EVI1 and synergistically induces megakaryocyte-characteristic leukemia with insufficient haploid dose of GATA2	(63)
MS4A3	One of the most strongly inhibited target genes of MECOM is achieved by directly binding to its promoter. Its down-regulation is crucial for MECOM-mediated tumorigenesis	(64)
ERG	The direct downstream targets of EVI1, activated by EVI1, are crucial for maintaining EVI1-driven leukemia	(65, 66)
Spi1 (PU.1)	1) transcriptionally activated, driving myeloid deviation; 2) Proteins are functionally inhibited by direct interactions, blocking terminal differentiation	(67, 68)
CtBP	EVI1 recruits key corepressors to form powerful transcriptional inhibitory complexes	(55)

Beyond histone modifications, EVI1 has been shown to be a master regulator of the leukemic epigenome by directing aberrant patterns of DNA methylation. EVI1-high leukemias exhibit a distinct DNA hypermethylation signature, suggesting that EVI1 either recruits DNA methyltransferases to specific loci or represses genes involved in demethylation, thereby sculpting a pathogenic epigenetic landscape (69). Its oncogenic activity is also linked to its ability to repress the expression of the retinoblastoma (Rb) tumor suppressor gene, further disabling cell cycle control (70). In a more recently discovered mechanism, EVI1 has been shown to drive leukemogenesis through the aberrant transcriptional activation of the proto-oncogene ERG, highlighting its capacity to function as both a repressor and an activator (71). Through these widespread effects on the transcriptome and epigenome, EVI1 establishes and maintains the malignant state.

### 4.4 Cooperation with other oncogenic lesions

Leukemogenesis is a multi-step process, and EVI1 rarely acts alone. Its potent transforming activity is often realized through collaboration with other cooperating genetic mutations. EVI1 overexpression is a critical cooperating event in leukemias driven by MLL rearrangements, such as MLL-AF9. In this context, EVI1 is essential for the maintenance and growth of the leukemic clone, and its expression is required for the full transforming potential of the MLL fusion protein (72, 73).

Similarly, EVI1 cooperates powerfully with mutations in AML1 (RUNX1). Mouse models have demonstrated that the combination of Aml1 mutation and Evi1 overexpression is sufficient to induce a disease that faithfully recapitulates human MDS/AML (74, 75). This synergy is also observed in the progression of familial platelet disorder (FPD) with propensity to AML, a hereditary syndrome caused by germline RUNX1 mutations, where secondary acquisition of EVI1 overexpression is a key step in leukemic transformation (76).

EVI1 also synergizes with other oncogenic drivers. It has been shown to cooperate with a truncated, pro-leukemogenic isoform of C/EBPβ (LIP) to induce AML (77). It collaborates with the transcription factor Sox4 to activate retroviral LTRs and promote leukemogenesis (78). In concert with Trib1, EVI1 can accelerate AML development in the context of Hoxa9/Meis1 overexpression (79). Furthermore, EVI1 overexpression frequently co-occurs with activating mutations in the RAS signaling pathway, particularly in inv(3)/t(3;3) leukemias, suggesting a strong synergy between these two oncogenic pathways (80). The combination of oncogenic Nras and Evi1 has been shown to be a potent driver of aggressive leukemia in mouse models (81). This extensive network of collaborations underscores EVI1’s role as a potent and versatile oncogenic hub.

## 5 Clinical and therapeutic implications of EVI1 expression

The profound biological effects of EVI1 translate directly into significant clinical consequences, establishing it as a critical factor in patient risk stratification and a prime candidate for targeted therapy.

### 5.1 EVI1 as a potent negative prognostic marker

Across numerous studies and diverse subtypes of hematological malignancies, high EVI1 expression is consistently and independently associated with a dismal clinical outcome. In AML, EVI1 overexpression is one of the most significant adverse prognostic markers, predicting primary refractory disease, high rates of relapse, and poor overall survival (11, 82). This holds true for both pediatric and adult AML (10). Even within specific genetic subgroups, such as AML with MLL rearrangements, co-expression of EVI1 delineates a subset of patients with a particularly poor prognosis (83). EVI1-rearranged AMLs are recognized as a distinct entity characterized by a unique mutational and transcriptional signature and an exceptionally aggressive clinical course (84).

In the context of CML, EVI1 expression is rarely detected in the chronic phase but becomes significantly upregulated during progression to the myeloid blast crisis, where it is implicated as a key driver of transformation (85). Moreover, in CML patients who have developed resistance to tyrosine kinase inhibitors like imatinib, high EVI1 expression is a predictor of poor survival (86). Its expression is also linked to adverse outcomes in MDS and has been found to be upregulated in chronic lymphocytic leukemia (CLL), where it contributes to cell survival by modulating microRNA networks (87). The consistent association of EVI1 with aggressive disease across different malignancies solidifies its status as a top-tier negative prognostic biomarker.

### 5.2 EVI1 as a therapeutic target

Given its critical role in driving leukemogenesis and its association with therapy resistance, EVI1 itself represents a high-priority therapeutic target (**Table 4**). Recent studies have proposed several strategies to therapeutically target EVI1, including degradation of the EVI1 protein via the ubiquitin-proteasome pathway, inhibition of its transcriptional co-factors such as CTBP1/2 and HDACs, and interference with upstream regulators that control EVI1 expression or stability.

**Table 4:** Summary of therapeutic strategies targeting EVI1-driven cancers.

Strategy/Drug category	Mechanism and effect	Reference
BET inhibitors (BETi)	Inhibit the key protein BRD4 of the super enhancer (SE), thereby down-regulating the expression of oncogenes such as EVI1 and MYC, and inducing apoptosis and differentiation of cells	(2, 29, 98)
HDAC inhibitors (HDACi)	Disrupt the MECOM co-inhibitory complex and directly inhibit the transcriptional and protein levels of MECOM. Targeting the co-transcription factor PA2G4 is also one of its mechanisms of HDACi	(90, 99)
Jumonji demethylase inhibitors (such as JIB-04)	By altering the H3K27me3 modification status of the MECOM promoter region, the expression of MECOM can be directly inhibited at the transcriptional level, which is effective for ovarian cancer	(100)
PARP inhibitors (PARPi)	Targeting PARP1, a core member of the super enhancer (SE) that drives MECOM expression, disrupts the structural integrity and chromatin loop of SE, thereby down-regulating MECOM expression	(101, 102)
Protein synthesis inhibitor (Homoharringtonine, HHT)	By inhibiting protein synthesis and indirectly down-regulating the levels of key oncoproteins such as EVI1 and c-Myc	(76)
MEK inhibitors	Indirectly inhibiting the downstream ERK/ZEB1 signaling pathway activated by the upregulation of KRAS expression by MECOM is applicable to specific ovarian cancers	(100)
PI3K/mTOR inhibitors (such as dactolisib)	Antagonize the continuous activation of the PI3K/AKT/mTOR pathway caused by MECOM’s inhibition of the tumor suppressor gene PTEN	(57)
Target SOX2 and cancer stemness	By directly activating the dry factor SOX2 as a transcription factor, MECOM inhibits the characteristics of tumor stem cells and is applicable to lung squamous cell carcinoma	(103)

Arsenic trioxide (ATO), a drug used in the treatment of acute promyelocytic leukemia (APL), has been shown to induce the degradation of the EVI1 oncoprotein, leading to cell cycle arrest and apoptosis in EVI1-positive leukemia cells (88). ATO can also induce autophagy-related cell death in these cells by downregulating EVI1 (89). These findings suggest a potential repurposing of ATO for EVI1-driven malignancies.

More recent strategies focus on targeting the epigenetic machinery that EVI1 relies upon. BET bromodomain inhibitors, which disrupt the function of transcriptional co-activators, have been shown to suppress EVI1 expression and induce apoptosis in EVI1-high AML cells (66). Similarly, histone deacetylase (HDAC) inhibitors have demonstrated efficacy by downregulating EVI1 expression through a mechanism involving the up-regulation of its repressor, PA2G4 (90). The dependency of EVI1-high cells on specific metabolic pathways, such as those regulated by PRMT5, also offers a potential therapeutic vulnerability (91).

### 5.3 EVI1 downstream effectors as potential therapeutic targets

Direct inhibition of EVI1 has historically been challenging due to its nature as a transcriptional regulator lacking enzymatic activity. Consequently, several indirect strategies have shown promise in suppressing EVI1-driven leukemogenesis. Such approaches collectively aim to dismantle the oncogenic transcriptional network sustained by EVI1 rather than targeting the protein itself.

A key target gene of EVI1 in HSCs is GATA-2, a transcription factor crucial for HSC proliferation. EVI1 directly binds to the GATA-2 promoter and acts as an enhancer, upregulating its expression. This EVI1-GATA-2 axis is fundamental for the maintenance and proliferation of HSCs. The reduction in GATA-2 expression in *Evi1*^-/-^ HSCs underscores the hierarchical regulation of the HSC pool by transcription factors (92).

Furthermore, EVI1 can repress the activity of RUNX1, a critical hematopoietic regulator. This repression can impair RUNX1’s ability to bind DNA and regulate gene expression, potentially contributing to leukemogenesis by disrupting normal hematopoietic programs. The eighth zinc finger motif of EVI1 is involved in this interaction, and its expression alone can block granulocyte differentiation, leading to cell death. This suggests that inappropriate EVI1 expression can contribute to hematopoietic transformation by functionally impairing key hematopoietic regulators (48).

In the context of myeloid malignancies, EVI1 can inhibit the expression of the membrane-spanning-4-domains subfamily-A member-3 (MS4A3) gene. MS4A3 has been implicated in promoting differentiation in chronic myeloid leukemia (CML) by enhancing common β-chain cytokine receptor endocytosis. Low MS4A3 expression is characteristic of LSPC quiescence and transformation to blast phase CML, and EVI1’s suppression of MS4A3 contributes to the differentiation block observed in CML. Promoting MS4A3 re-expression or delivery of ectopic MS4A3 may be a therapeutic strategy to eliminate CML stem cells (93, 94).

Another approach involves targeting surrogate surface markers. It has been discovered that EVI1-high AML cells frequently express high levels of the surface protein CD52, which is not typically found on normal myeloid progenitors. This makes CD52 a potential target for antibody-based immunotherapy, such as with alemtuzumab, to specifically eliminate the EVI1-positive leukemic clone (95). Paradoxically, some studies have suggested that EVI1-positive AML cells may exhibit sensitivity to all-trans retinoic acid (ATRA), a differentiating agent, potentially through EVI1’s regulation by retinoic acid receptors (96, 97). These diverse approaches highlight the active search for effective therapies to combat these aggressive leukemias.

## 6 Conclusion

Collectively, EVI1 act as a central regulator under both physiological and pathological conditions. Under normal homeostasis, EVI1 plays an essential role in maintaining HSC self-renewal and long-term integrity (9, 104), and is also critically involved in endothelial lineage specification and broader developmental programs (105). In contrast, under pathological conditions, aberrant overexpression of EVI1, functions as a potent oncogenic driver across a wide spectrum of malignancies, including AML, MDS, lymphoid leukemias (87, 106), as well as several solid tumors such as ovarian and prostate cancers (107, 108).

In hematologic malignancies, high EVI1 expression has been established as an independent adverse prognostic biomarker. Regardless of the presence of classical 3q26 chromosomal abnormalities, elevated EVI1 levels are consistently associated with chemoresistance, increased relapse risk, and markedly inferior overall survival (11, 109). As a master transcription factor, EVI1 profoundly rewires the cellular machinery of hematopoietic progenitors. It potently blocks myeloid differentiation by antagonizing essential lineage-defining factors like RUNX1, while simultaneously promoting cell survival and proliferation through the subversion of critical signaling pathways, most notably by disabling the tumor-suppressive TGF-β pathway and activating the pro-survival PI3K/AKT/mTOR cascade.

Despite substantial progress in understanding EVI1 biology, several fundamental questions remain unresolved. Notably, EVI1 exhibits pronounced context-dependent functionality, displaying both oncogenic and tumor-suppressive features. On one hand, EVI1 acts as a potent oncogenic driver by enforcing differentiation blockade and promoting leukemic proliferation. On the other hand, its haploinsufficiency leads to bone marrow failure syndromes characterized by congenital amegakaryocytic thrombocytopenia, underscoring its essential role in maintaining hematopoietic homeostasis (110). Moreover, in certain MDS subtypes, low-level EVI1 expression does not appear to represent a primary driver of ineffective hematopoiesis, suggesting the presence of dosage-dependent effects and strong cell-context specificity in EVI1 function.

In addition, the precise mechanisms governing EVI1 transcriptional regulation remain elusive. Classical 3q26 rearrangements, such as inv(3)(q21q26), induce aberrant EVI1 activation through an enhancer hijacking mechanism, whereby distal regulatory elements, including the GATA2 enhancer, are repositioned in proximity to the EVI1 promoter, resulting in constitutive overexpression (29). However, in cases lacking 3q26 abnormalities, the upstream regulatory circuitry controlling EVI1 expression appears even more heterogeneous, involving multiple transcription factors and signaling pathways. For instance, MECOM expression has been shown to be modulated by proteins such as Survivin (111). Downstream, EVI1 binds distinct DNA motifs through its two independent zinc finger domains, thereby regulating a broad repertoire of cancer-associated target genes (107), often in cooperation with transcription factors such as AP-1/FOS. In addition, EVI1 directly regulates PLZF (53) and multiple microRNAs, including miR-9 (112, 113), collectively establishing a multilayered transcriptional and epigenetic regulatory network.

Looking ahead, a deeper understanding of the context-dependent functional plasticity of EVI1 across physiological and malignant states will be critical for translating mechanistic insights into therapeutic advances. Dissecting subtype-specific EVI1 activities and constructing comprehensive regulatory networks that integrate transcriptional, epigenetic, and signaling pathways will help identify actionable dependencies in EVI1-driven malignancies. Emerging technologies, including single-cell multi-omics and *in vivo* functional modeling within the native hematopoietic niche, offer powerful platforms to define how EVI1 governs stem cell fate and therapy resistance in real time (114, 115). Importantly, systematic interrogation of EVI1-centered regulatory circuits is expected to reveal targetable vulnerabilities. Therapeutic strategies aimed at disrupting EVI1 activation, its essential cofactors, or key downstream effectors may provide a rational framework for precision treatment of EVI1-high acute myeloid leukemia and myelodysplastic syndromes, addressing an urgent unmet clinical need in these high-risk diseases.

## References

[1] Mucenski ML Taylor BA Ihle JN Hartley JW Morse HC 3rd. Jenkins NA . Identification of a common ecotropic viral integration site, Evi-1, in the DNA of AKXD murine myeloid tumors. Mol Cell Biol. (1988) 8:301–8. doi: 10.1128/mcb.8.1.301-308.1988, PMID: 2827004 PMC363121

[2] Groschel S Sanders MA Hoogenboezem R de Wit E Bouwman BAM Erpelinck C . A single oncogenic enhancer rearrangement causes concomitant EVI1 and GATA2 deregulation in leukemia. Cell. (2014) 157:369–81. doi: 10.1016/j.cell.2014.02.019, PMID: 24703711

[3] Du Y Jenkins NA Copeland NG . Insertional mutagenesis identifies genes that promote the immortalization of primary bone marrow progenitor cells. Blood. (2005) 106:3932–9. doi: 10.1182/blood-2005-03-1113, PMID: 16109773 PMC1895096

[4] Russell M List A Greenberg P Woodward S Glinsmann B Parganas E . Expression of EVI1 in myelodysplastic syndromes and other hematologic Malignancies without 3q26 translocations. Blood. (1994) 84:1243–8. doi: 10.1182/blood.V84.4.1243.1243, PMID: 8049440

[5] Tanaka A Nakano TA Nomura M Yamazaki H Bewersdorf JP Mulet-Lazaro R . Aberrant EVI1 splicing contributes to EVI1-rearranged leukemia. Blood. (2022) 140:875–88. doi: 10.1182/blood.2021015325, PMID: 35709354 PMC9412007

[6] Delwel R Funabiki T Kreider BL Morishita K Ihle JN . Four of the seven zinc fingers of the Evi-1 myeloid-transforming gene are required for sequence-specific binding to GA(C/T)AAGA(T/C)AAGATAA. Mol Cell Biol. (1993) 13:4291–300. doi: 10.1128/mcb.13.7.4291-4300.1993, PMID: 8321231 PMC359982

[7] Pastoors D Havermans M Mulet-Lazaro R Smeenk L Ottema S Erpelinck-Verschueren CAJ . MECOM is a master repressor of myeloid differentiation through dose control of CEBPA in acute myeloid leukemia. Blood. (2025) 146(25):3098–105. doi: 10.1182/blood.2025028914, PMID: 41060369

[8] Maicas M Vazquez I Alis R Marcotegui N Urquiza L Cortes-Lavaud X . The MDS and EVI1 complex locus (MECOM) isoforms regulate their own transcription and have different roles in the transformation of hematopoietic stem and progenitor cells. Biochim Biophys Acta Gene Regul Mech. (2017) 1860:721–9. doi: 10.1016/j.bbagrm.2017.03.007, PMID: 28391050

[9] Goyama S Yamamoto G Shimabe M Sato T Ichikawa M Ogawa S . Evi-1 is a critical regulator for hematopoietic stem cells and transformed leukemic cells. Cell Stem Cell. (2008) 3:207–20. doi: 10.1016/j.stem.2008.06.002, PMID: 18682242

[10] Balgobind BV Lugthart S Hollink IH Arentsen-Peters ST van Wering ER de Graaf SS . EVI1 overexpression in distinct subtypes of pediatric acute myeloid leukemia. Leukemia. (2010) 24:942–9. doi: 10.1038/leu.2010.47, PMID: 20357826

[11] Santamaria CM Chillon MC Garcia-Sanz R Perez C Caballero MD Ramos F . Molecular stratification model for prognosis in cytogenetically normal acute myeloid leukemia. Blood. (2009) 114:148–52. doi: 10.1182/blood-2008-11-187724, PMID: 19398719

[12] Cai SF Chu SH Goldberg AD Parvin S Koche RP Glass JL . Leukemia cell of origin influences apoptotic priming and sensitivity to LSD1 inhibition. Cancer Discov. (2020) 10:1500–13. doi: 10.1158/2159-8290.CD-19-1469, PMID: 32606137 PMC7584353

[13] Kurokawa M Mitani K Irie K Matsuyama T Takahashi T Chiba S . The oncoprotein Evi-1 represses TGF-beta signalling by inhibiting Smad3. Nature. (1998) 394:92–6. doi: 10.1038/27945, PMID: 9665135

[14] Nagai K Niihori T Muto A Hayashi Y Abe T Igarashi K . Mecom mutation related to radioulnar synostosis with amegakaryocytic thrombocytopenia reduces HSPCs in mice. Blood Adv. (2023) 7:5409–20. doi: 10.1182/bloodadvances.2022008462, PMID: 37099686 PMC10509669

[15] Ivanochko D Halabelian L Henderson E Savitsky P Jain H Marcon E . Direct interaction between the PRDM3 and PRDM16 tumor suppressors and the NuRD chromatin remodeling complex. Nucleic Acids Res. (2019) 47:1225–38. doi: 10.1093/nar/gky1192, PMID: 30462309 PMC6379669

[16] Soderholm J Kobayashi H Mathieu C Rowley JD Nucifora G . The leukemia-associated gene MDS1/EVI1 is a new type of GATA-binding transactivator. Leukemia. (1997) 11:352–8. doi: 10.1038/sj.leu.2400584, PMID: 9067573

[17] Sood R Talwar-Trikha A Chakrabarti SR Nucifora G . MDS1/EVI1 enhances TGF-beta1 signaling and strengthens its growth-inhibitory effect but the leukemia-associated fusion protein AML1/MDS1/EVI1, product of the t(3;21), abrogates growth-inhibition in response to TGF-beta1. Leukemia. (1999) 13:348–57. doi: 10.1038/sj.leu.2401360, PMID: 10086725

[18] Mochizuki N Shimizu S Nagasawa T Tanaka H Taniwaki M Yokota J . A novel gene, MEL1, mapped to 1p36.3 is highly homologous to the MDS1/EVI1 gene and is transcriptionally activated in t(1;3)(p36;q21)-positive leukemia cells. Blood. (2000) 96:3209–14. doi: 10.1182/blood.V96.9.3209, PMID: 11050005

[19] Nishikata I Sasaki H Iga M Tateno Y Imayoshi S Asou N . A novel EVI1 gene family, MEL1, lacking a PR domain (MEL1S) is expressed mainly in t(1;3)(p36;q21)-positive AML and blocks G-CSF-induced myeloid differentiation. Blood. (2003) 102:3323–32. doi: 10.1182/blood-2002-12-3944, PMID: 12816872

[20] Lugthart S Groschel S Beverloo HB Kayser S Valk PJ van Zelderen-Bhola SL . Clinical, molecular, and prognostic significance of WHO type inv(3)(q21q26.2)/t(3;3)(q21;q26.2) and various other 3q abnormalities in acute myeloid leukemia. J Clin Oncol. (2010) 28:3890–8. doi: 10.1200/JCO.2010.29.2771, PMID: 20660833

[21] Shearer BM Sukov WR Flynn HC Knudson RA Ketterling RP . Development of a dual-color, double fusion FISH assay to detect RPN1/EVI1 gene fusion associated with inv(3), t(3;3), and ins(3;3) in patients with myelodysplasia and acute myeloid leukemia. Am J Hematol. (2010) 85:569–74. doi: 10.1002/ajh.21746, PMID: 20556821

[22] Zhu YM Wang PP Huang JY Chen YS Chen B Dai YJ . Gene mutational pattern and expression level in 560 acute myeloid leukemia patients and their clinical relevance. J Transl Med. (2017) 15:178. doi: 10.1186/s12967-017-1279-4, PMID: 28830460 PMC5568401

[23] Pastoors D Havermans M Mulet-Lazaro R Brian D Noort W Grasel J . Oncogene EVI1 drives acute myeloid leukemia via a targetable interaction with CTBP2. Sci Adv. (2024) 10:eadk9076. doi: 10.1126/sciadv.adk9076, PMID: 38748792 PMC11095456

[24] Buonamici S Li D Chi Y Zhao R Wang X Brace L . EVI1 induces myelodysplastic syndrome in mice. J Clin Invest. (2004) 114:713–9. doi: 10.1172/JCI21716, PMID: 15343390 PMC514587

[25] Jimenez-Vicente C Esteve J Baile-Gonzalez M Perez-Lopez E Martin Calvo C Aparicio C . Allo-HCT refined ELN 2022 risk classification: validation of the Adverse-Plus risk group in AML patients undergoing allogeneic hematopoietic cell transplantation within the Spanish Group for Hematopoietic Cell Transplantation (GETH-TC). Blood Cancer J. (2025) 15:42. doi: 10.1038/s41408-025-01223-x, PMID: 40118819 PMC11928450

[26] Jones D Luthra R Cortes J Thomas D O’Brien S Bueso-Ramos C . BCR-ABL fusion transcript types and levels and their interaction with secondary genetic changes in determining the phenotype of Philadelphia chromosome-positive leukemias. Blood. (2008) 112:5190–2. doi: 10.1182/blood-2008-04-148791, PMID: 18809762 PMC2597614

[27] Smeenk L Ottema S Mulet-Lazaro R Ebert A Havermans M Varea AA . Selective requirement of MYB for oncogenic hyperactivation of a translocated enhancer in leukemia. Cancer Discov. (2021) 11:2868–83. doi: 10.1158/2159-8290.CD-20-1793, PMID: 33980539 PMC8563373

[28] Koche RP Armstrong SA . Genomic dark matter sheds light on EVI1-driven leukemia. Cancer Cell. (2014) 25:407–8. doi: 10.1016/j.ccr.2014.03.031, PMID: 24735919 PMC4046890

[29] Yamazaki H Suzuki M Otsuki A Shimizu R Bresnick EH Engel JD . A remote GATA2 hematopoietic enhancer drives leukemogenesis in inv(3)(q21;q26) by activating EVI1 expression. Cancer Cell. (2014) 25:415–27. doi: 10.1016/j.ccr.2014.02.008, PMID: 24703906 PMC4012341

[30] Johnson KD Kong G Gao X Chang YI Hewitt KJ Sanalkumar R . Cis-regulatory mechanisms governing stem and progenitor cell transitions. Sci Adv. (2015) 1:e1500503. doi: 10.1126/sciadv.1500503, PMID: 26601269 PMC4643771

[31] Ottema S Mulet-Lazaro R Beverloo HB Erpelinck C van Herk S van der Helm R . Atypical 3q26/MECOM rearrangements genocopy inv(3)/t(3;3) in acute myeloid leukemia. Blood. (2020) 136:224–34. doi: 10.1182/blood.2019003701, PMID: 32219447

[32] Nakamura F Nakamura Y Sasaki K Yamazaki I Imai Y Mitani K . HDAC inhibitors repress Tek and Angpt1 expression and proliferation in RUNX1-MECOM-type leukemia cells. Leuk Res. (2025) 156:107738. doi: 10.1016/j.leukres.2025.107738, PMID: 40570741

[33] Maki K Yamagata T Asai T Yamazaki I Oda H Hirai H . Dysplastic definitive hematopoiesis in AML1/EVI1 knock-in embryos. Blood. (2005) 106:2147–55. doi: 10.1182/blood-2004-11-4330, PMID: 15914564

[34] Nakamura Y Ichikawa M Oda H Yamazaki I Sasaki K Mitani K . RUNX1-EVI1 induces dysplastic hematopoiesis and acute leukemia of the megakaryocytic lineage in mice. Leuk Res. (2018) 74:14–20. doi: 10.1016/j.leukres.2018.09.015, PMID: 30278283

[35] Ronaghy A Hu S Tang Z Wang W Tang G Loghavi S . Myeloid neoplasms associated with t(3;12)(q26.2;p13) are clinically aggressive, show myelodysplasia, and frequently harbor chromosome 7 abnormalities. Mod Pathol. (2021) 34:300–13. doi: 10.1038/s41379-020-00663-z, PMID: 33110238

[36] Ottema S Mulet-Lazaro R Erpelinck-Verschueren C van Herk S Havermans M Arricibita Varea A . The leukemic oncogene EVI1 hijacks a MYC super-enhancer by CTCF-facilitated loops. Nat Commun. (2021) 12:5679. doi: 10.1038/s41467-021-25862-3, PMID: 34584081 PMC8479123

[37] Trubia M Albano F Cavazzini F Cambrin GR Quarta G Fabbiano F . Characterization of a recurrent translocation t(2;3)(p15-22;q26) occurring in acute myeloid leukaemia. Leukemia. (2006) 20:48–54. doi: 10.1038/sj.leu.2404020, PMID: 16619048

[38] Mitani K Ogawa S Tanaka T Miyoshi H Kurokawa M Mano H . Generation of the AML1-EVI-1 fusion gene in the t(3;21)(q26;q22) causes blastic crisis in chronic myelocytic leukemia. EMBO J. (1994) 13:504–10. doi: 10.1002/j.1460-2075.1994.tb06288.x, PMID: 8313895 PMC394839

[39] Tokita K Maki K Mitani K . RUNX1/EVI1, which blocks myeloid differentiation, inhibits CCAAT-enhancer binding protein alpha function. Cancer Sci. (2007) 98:1752–7. doi: 10.1111/j.1349-7006.2007.00597.x, PMID: 17894555 PMC11158720

[40] Takeshita M Ichikawa M Nitta E Goyama S Asai T Ogawa S . AML1-Evi-1 specifically transforms hematopoietic stem cells through fusion of the entire Evi-1 sequence to AML1. Leukemia. (2008) 22:1241–9. doi: 10.1038/leu.2008.53, PMID: 18337762

[41] Tanaka K Tanaka T Kurokawa M Imai Y Ogawa S Mitani K . The AML1/ETO(MTG8) and AML1/evi-1 leukemia-associated chimeric oncoproteins accumulate PEBP2β(CBFβ) in the nucleus more efficiently than wild-type AML1. Blood. (1998) 91:1688–99. doi: 10.1182/blood.V91.5.1688, PMID: 9473235

[42] Raynaud SD Baens M Grosgeorge J Rodgers K Reid CD Dainton M . Fluorescence in *situ* hybridization analysis of t(3; 12)(q26; p13): a recurring chromosomal abnormality involving the TEL gene (ETV6) in myelodysplastic syndromes. Blood. (1996) 88:682–9. doi: 10.1182/blood.V88.2.682.bloodjournal882682, PMID: 8695816

[43] Nakamura Y Nakazato H Sato Y Furusawa S Mitani K . Expression of the TEL/EVI1 fusion transcript in a patient with chronic myelogenous leukemia with t(3;12)(q26;p13). Am J Hematol. (2002) 69:80–2. doi: 10.1002/ajh.10028, PMID: 11835339

[44] Suzukawa K Kodera T Shimizu S Nagasawa T Asou H Kamada N . Activation of EVI1 transcripts with chromosomal translocation joining the TCRVbeta locus and the EVI1 gene in human acute undifferentiated leukemia cell line (Kasumi-3) with a complex translocation of der(3)t(3;7;8). Leukemia. (1999) 13:1359–66. doi: 10.1038/sj.leu.2401483, PMID: 10482986

[45] Arindrarto W Borras DM de Groen RAL van den Berg RR Locher IJ van Diessen S . Comprehensive diagnostics of acute myeloid leukemia by whole transcriptome RNA sequencing. Leukemia. (2021) 35:47–61. doi: 10.1038/s41375-020-0762-8, PMID: 32127641 PMC7787979

[46] Modlich U Schambach A Brugman MH Wicke DC Knoess S Li Z . Leukemia induction after a single retroviral vector insertion in Evi1 or Prdm16. Leukemia. (2008) 22:1519–28. doi: 10.1038/leu.2008.118, PMID: 18496560

[47] Bosticardo M Ghosh A Du Y Jenkins NA Copeland NG Candotti F . Self-inactivating retroviral vector-mediated gene transfer induces oncogene activation and immortalization of primary murine bone marrow cells. Mol Ther. (2009) 17:1910–8. doi: 10.1038/mt.2009.172, PMID: 19638958 PMC2835037

[48] Senyuk V Sinha KK Li D Rinaldi CR Yanamandra S Nucifora G . Repression of RUNX1 activity by EVI1: a new role of EVI1 in leukemogenesis. Cancer Res. (2007) 67:5658–66. doi: 10.1158/0008-5472.CAN-06-3962, PMID: 17575132

[49] Sitailo S Sood R Barton K Nucifora G . Forced expression of the leukemia-associated gene EVI1 in ES cells: a model for myeloid leukemia with 3q26 rearrangements. Leukemia. (1999) 13:1639–45. doi: 10.1038/sj.leu.2401585, PMID: 10557037

[50] Nishikawa S Arai S Masamoto Y Kagoya Y Toya T Watanabe-Okochi N . Thrombopoietin/MPL signaling confers growth and survival capacity to CD41-positive cells in a mouse model of Evi1 leukemia. Blood. (2014) 124:3587–96. doi: 10.1182/blood-2013-12-546275, PMID: 25298035

[51] Saito Y Kaneda K Suekane A Ichihara E Nakahata S Yamakawa N . Maintenance of the hematopoietic stem cell pool in bone marrow niches by EVI1-regulated GPR56. Leukemia. (2013) 27:1637–49. doi: 10.1038/leu.2013.75, PMID: 23478665

[52] Ohyashiki JH Ohyashiki K Shimamoto T Kawakubo K Fujimura T Nakazawa S . Ecotropic virus integration site-1 gene preferentially expressed in post-myelodysplasia acute myeloid leukemia: possible association with GATA-1, GATA-2, and stem cell leukemia gene expression. Blood. (1995) 85:3713–8. doi: 10.1182/blood.V85.12.3713.bloodjournal85123713, PMID: 7780155

[53] Takahashi S Licht JD . The human promyelocytic leukemia zinc finger gene is regulated by the Evi-1 oncoprotein and a novel guanine-rich site binding protein. Leukemia. (2002) 16:1755–62. doi: 10.1038/sj.leu.2402682, PMID: 12200691

[54] Kurokawa M Mitani K Imai Y Ogawa S Yazaki Y Hirai H . The t(3;21) fusion product, AML1/evi-1, interacts with smad3 and blocks transforming growth factor-β–mediated growth inhibition of myeloid cells. Blood. (1998) 92:4003–12. doi: 10.1182/blood.V92.11.4003, PMID: 9834202

[55] Izutsu K Kurokawa M Imai Y Maki K Mitani K Hirai H . The corepressor CtBP interacts with Evi-1 to repress transforming growth factor beta signaling. Blood. (2001) 97:2815–22. doi: 10.1182/blood.v97.9.2815, PMID: 11313276

[56] Paredes R Schneider M Stevens A White DJ Williamson AJK Muter J . EVI1 carboxy-terminal phosphorylation is ATM-mediated and sustains transcriptional modulation and self-renewal via enhanced CtBP1 association. Nucleic Acids Res. (2018) 46:7662–74. doi: 10.1093/nar/gky536, PMID: 29939287 PMC6125627

[57] Yoshimi A Goyama S Watanabe-Okochi N Yoshiki Y Nannya Y Nitta E . Evi1 represses PTEN expression and activates PI3K/AKT/mTOR via interactions with polycomb proteins. Blood. (2011) 117:3617–28. doi: 10.1182/blood-2009-12-261602, PMID: 21289308

[58] Wu Q Yu C Yu F Guo Y Sheng Y Li L . Evi1 governs Kdm6b-mediated histone demethylation to regulate the Laptm4b-driven mTOR pathway in hematopoietic progenitor cells. J Clin Invest. (2024) 134:e173403. doi: 10.1172/JCI173403, PMID: 39680456 PMC11645144

[59] Goyama S Nitta E Yoshino T Kako S Watanabe-Okochi N Shimabe M . EVI-1 interacts with histone methyltransferases SUV39H1 and G9a for transcriptional repression and bone marrow immortalization. Leukemia. (2010) 24:81–8. doi: 10.1038/leu.2009.202, PMID: 19776757

[60] Fenouille N Bassil CF Ben-Sahra I Benajiba L Alexe G Ramos A . The creatine kinase pathway is a metabolic vulnerability in EVI1-positive acute myeloid leukemia. Nat Med. (2017) 23:301–13. doi: 10.1038/nm.4283, PMID: 28191887 PMC5540325

[61] Kreider BL Orkin SH Ihle JN . Loss of erythropoietin responsiveness in erythroid progenitors due to expression of the Evi-1 myeloid-transforming gene. Proc Natl Acad Sci U.S.A. (1993) 90:6454–8. doi: 10.1073/pnas.90.14.6454, PMID: 8341654 PMC46950

[62] Dickstein J Senyuk V Premanand K Laricchia-Robbio L Xu P Cattaneo F . Methylation and silencing of miRNA-124 by EVI1 and self-renewal exhaustion of hematopoietic stem cells in murine myelodysplastic syndrome. Proc Natl Acad Sci U.S.A. (2010) 107:9783–8. doi: 10.1073/pnas.1004297107, PMID: 20448201 PMC2906858

[63] Yamaoka A Suzuki M Katayama S Orihara D Engel JD Yamamoto M . EVI1 and GATA2 misexpression induced by inv(3)(q21q26) contribute to megakaryocyte-lineage skewing and leukemogenesis. Blood Adv. (2020) 4:1722–36. doi: 10.1182/bloodadvances.2019000978, PMID: 32330245 PMC7189294

[64] Heller G Rommer A Steinleitner K Etzler J Hackl H Heffeter P . EVI1 promotes tumor growth via transcriptional repression of MS4A3. J Hematol Oncol. (2015) 8:28. doi: 10.1186/s13045-015-0124-6, PMID: 25886616 PMC4389965

[65] Du W He J Zhou W Shu S Li J Liu W . High IL2RA mRNA expression is an independent adverse prognostic biomarker in core binding factor and intermediate-risk acute myeloid leukemia. J Transl Med. (2019) 17:191. doi: 10.1186/s12967-019-1926-z, PMID: 31171000 PMC6551869

[66] Birdwell CE Fiskus W Kadia TM Mill CP Sasaki K Daver N . Preclinical efficacy of targeting epigenetic mechanisms in AML with 3q26 lesions and EVI1 overexpression. Leukemia. (2024) 38:545–56. doi: 10.1038/s41375-023-02108-3, PMID: 38086946 PMC12122007

[67] Ayoub E Wilson MP McGrath KE Li AJ Frisch BJ Palis J . EVI1 overexpression reprograms hematopoiesis via upregulation of Spi1 transcription. Nat Commun. (2018) 9:4239. doi: 10.1038/s41467-018-06208-y, PMID: 30315161 PMC6185954

[68] Laricchia-Robbio L Premanand K Rinaldi CR Nucifora G . EVI1 Impairs myelopoiesis by deregulation of PU.1 function. Cancer Res. (2009) 69:1633–42. doi: 10.1158/0008-5472.CAN-08-2562, PMID: 19208846

[69] Lugthart S Figueroa ME Bindels E Skrabanek L Valk PJ Li Y . Aberrant DNA hypermethylation signature in acute myeloid leukemia directed by EVI1. Blood. (2011) 117:234–41. doi: 10.1182/blood-2010-04-281337, PMID: 20855866 PMC3037746

[70] Yufu Y Sadamura S Ishikura H Abe Y Katsuno M Nishimura J . Expression of EVI1 and the retinoblastoma genes in acute myelogenous leukemia with t(3;13)(q26;q13–14). Am J Hematol. (1996) 53:30–4. doi: 10.1002/(sici)1096-8652(199609)53:1<30::Aid-ajh6>3.0.Co;2-6, PMID: 8813093

[71] Schmoellerl J Barbosa IAM Minnich M Andersch F Smeenk L Havermans M . EVI1 drives leukemogenesis through aberrant ERG activation. Blood. (2023) 141:453–66. doi: 10.1182/blood.2022016592, PMID: 36095844

[72] Bindels EM Havermans M Lugthart S Erpelinck C Wocjtowicz E Krivtsov AV . EVI1 is critical for the pathogenesis of a subset of MLL-AF9-rearranged AMLs. Blood. (2012) 119:5838–49. doi: 10.1182/blood-2011-11-393827, PMID: 22553314 PMC3382941

[73] Zhang Y Owens K Hatem L Glass CH Karuppaiah K Camargo F . Essential role of PR-domain protein MDS1-EVI1 in MLL-AF9 leukemia. Blood. (2013) 122:2888–92. doi: 10.1182/blood-2012-08-453662, PMID: 24021671 PMC3799001

[74] Watanabe-Okochi N Kitaura J Ono R Harada H Harada Y Komeno Y . AML1 mutations induced MDS and MDS/AML in a mouse BMT model. Blood. (2008) 111:4297–308. doi: 10.1182/blood-2007-01-068346, PMID: 18192504

[75] Graubert T . AML1 and evi1: coconspirators in MDS/AML? Blood. (2008) 111:3916–7. doi: 10.1182/blood-2008-01-135376, PMID: 18434965

[76] Mill CP Fiskus WC DiNardo CD Reville P Davis JA Birdwell CE . Efficacy of novel agents against cellular models of familial platelet disorder with myeloid Malignancy (FPD-MM). Blood Cancer J. (2024) 14:25. doi: 10.1038/s41408-024-00981-4, PMID: 38316746 PMC10844204

[77] Watanabe-Okochi N Yoshimi A Sato T Ikeda T Kumano K Taoka K . The shortest isoform of C/EBPbeta, liver inhibitory protein (LIP), collaborates with Evi1 to induce AML in a mouse BMT model. Blood. (2013) 121:4142–55. doi: 10.1182/blood-2011-07-368654, PMID: 23547050

[78] Boyd KE Xiao YY Fan K Poholek A Copeland NG Jenkins NA . Sox4 cooperates with Evi1 in AKXD-23 myeloid tumors via transactivation of proviral LTR. Blood. (2006) 107:733–41. doi: 10.1182/blood-2003-05-1626, PMID: 16204320 PMC1895620

[79] Jin G Yamazaki Y Takuwa M Takahara T Kaneko K Kuwata T . Trib1 and Evi1 cooperate with Hoxa and Meis1 in myeloid leukemogenesis. Blood. (2007) 109:3998–4005. doi: 10.1182/blood-2006-08-041202, PMID: 17227832

[80] Groschel S Sanders MA Hoogenboezem R Zeilemaker A Havermans M Erpelinck C . Mutational spectrum of myeloid Malignancies with inv(3)/t(3;3) reveals a predominant involvement of RAS/RTK signaling pathways. Blood. (2015) 125:133–9. doi: 10.1182/blood-2014-07-591461, PMID: 25381062 PMC4334729

[81] Li Q Haigis KM McDaniel A Harding-Theobald E Kogan SC Akagi K . Hematopoiesis and leukemogenesis in mice expressing oncogenic NrasG12D from the endogenous locus. Blood. (2011) 117:2022–32. doi: 10.1182/blood-2010-04-280750, PMID: 21163920 PMC3056645

[82] Groschel S Lugthart S Schlenk RF Valk PJ Eiwen K Goudswaard C . High EVI1 expression predicts outcome in younger adult patients with acute myeloid leukemia and is associated with distinct cytogenetic abnormalities. J Clin Oncol. (2010) 28:2101–7. doi: 10.1200/JCO.2009.26.0646, PMID: 20308656

[83] Groschel S Schlenk RF Engelmann J Rockova V Teleanu V Kuhn MW . Deregulated expression of EVI1 defines a poor prognostic subset of MLL-rearranged acute myeloid leukemias: a study of the German-Austrian Acute Myeloid Leukemia Study Group and the Dutch-Belgian-Swiss HOVON/SAKK Cooperative Group. J Clin Oncol. (2013) 31:95–103. doi: 10.1200/JCO.2011.41.5505, PMID: 23008312

[84] Lavallee VP Gendron P Lemieux S D’Angelo G Hebert J Sauvageau G . EVI1-rearranged acute myeloid leukemias are characterized by distinct molecular alterations. Blood. (2015) 125:140–3. doi: 10.1182/blood-2014-07-591529, PMID: 25331116 PMC4358966

[85] De Weer A Poppe B Cauwelier B Carlier A Dierick J Verhasselt B . EVI1 activation in blast crisis CML due to juxtaposition to the rare 17q22 partner region as part of a 4-way variant translocation t(9;22). BMC Cancer. (2008) 8:193. doi: 10.1186/1471-2407-8-193, PMID: 18613965 PMC2474635

[86] Daghistani M Marin D Khorashad JS Wang L May PC Paliompeis C . EVI-1 oncogene expression predicts survival in chronic-phase CML patients resistant to imatinib treated with second-generation tyrosine kinase inhibitors. Blood. (2010) 116:6014–7. doi: 10.1182/blood-2010-01-264234, PMID: 20855863

[87] Vasyutina E Boucas JM Bloehdorn J Aszyk C Crispatzu G Stiefelhagen M . The regulatory interaction of EVI1 with the TCL1A oncogene impacts cell survival and clinical outcome in CLL. Leukemia. (2015) 29:2003–14. doi: 10.1038/leu.2015.114, PMID: 25936528

[88] Shackelford D Kenific C Blusztajn A Waxman S Ren R . Targeted degradation of the AML1/MDS1/EVI1 oncoprotein by arsenic trioxide. Cancer Res. (2006) 66:11360–9. doi: 10.1158/0008-5472.CAN-06-1774, PMID: 17145882

[89] Smith DM Patel S Raffoul F Haller E Mills GB Nanjundan M . Arsenic trioxide induces a beclin-1-independent autophagic pathway via modulation of SnoN/SkiL expression in ovarian carcinoma cells. Cell Death Differ. (2010) 17:1867–81. doi: 10.1038/cdd.2010.53, PMID: 20508647 PMC2932795

[90] Marchesini M Gherli A Simoncini E Tor LMD Montanaro A Thongon N . Orthogonal proteogenomic analysis identifies the druggable PA2G4-MYC axis in 3q26 AML. Nat Commun. (2024) 15:4739. doi: 10.1038/s41467-024-48953-3, PMID: 38834613 PMC11150407

[91] Szewczyk MM Luciani GM Vu V Murison A Dilworth D Barghout SH . PRMT5 regulates ATF4 transcript splicing and oxidative stress response. Redox Biol. (2022) 51:102282. doi: 10.1016/j.redox.2022.102282, PMID: 35305370 PMC8933703

[92] Yuasa H Oike Y Iwama A Nishikata I Sugiyama D Perkins A . Oncogenic transcription factor Evi1 regulates hematopoietic stem cell proliferation through GATA-2 expression. EMBO J. (2005) 24:1976–87. doi: 10.1038/sj.emboj.7600679, PMID: 15889140 PMC1142611

[93] Zhao H Pomicter AD Eiring AM Franzini A Ahmann J Hwang JY . MS4A3 promotes differentiation in chronic myeloid leukemia by enhancing common beta-chain cytokine receptor endocytosis. Blood. (2022) 139:761–78. doi: 10.1182/blood.2021011802, PMID: 34780648 PMC8814676

[94] Jiang M Zou X Huang W . Ecotropic viral integration site 1 regulates the progression of acute myeloid leukemia via MS4A3-mediated TGFbeta/EMT signaling pathway. Oncol Lett. (2018) 16:2701–8. doi: 10.3892/ol.2018.8890, PMID: 30013666 PMC6036571

[95] Saito Y Nakahata S Yamakawa N Kaneda K Ichihara E Suekane A . CD52 as a molecular target for immunotherapy to treat acute myeloid leukemia with high EVI1 expression. Leukemia. (2011) 25:921–31. doi: 10.1038/leu.2011.36, PMID: 21394097

[96] Verhagen HJ Smit MA Rutten A Denkers F Poddighe PJ Merle PA . Primary acute myeloid leukemia cells with overexpression of EVI-1 are sensitive to all-trans retinoic acid. Blood. (2016) 127:458–63. doi: 10.1182/blood-2015-07-653840, PMID: 26582376

[97] Xi ZF Russell M Woodward S Thompson F Wagner L Taetle R . Expression of the Zn finger gene, EVI-1, in acute promyelocytic leukemia. Leukemia. (1997) 11:212–20. doi: 10.1038/sj.leu.2400547, PMID: 9009083

[98] Birdwell CE Fiskus W Mill CP Kadia TM Daver N DiNardo CD . BET inhibitor-based combinations targeting novel dependencies in MECOM-rearranged (r) AML. Leukemia. (2025) 40(2):304–13. doi: 10.1038/s41375-025-02842-w, PMID: 41419608 PMC12875863

[99] Vinatzer U Taplick J Seiser C Fonatsch C Wieser R . The leukaemia-associated transcription factors EVI-1 and MDS1/EVI1 repress transcription and interact with histone deacetylase. Br J Haematol. (2001) 114:566–73. doi: 10.1046/j.1365-2141.2001.02987.x, PMID: 11552981

[100] Singh I Karna A Prajapati A Solanki U Mukherjee A Uppal S . Epigenetic targeting of MECOM/KRAS axis by JIB-04 impairs tumorigenesis and cisplatin resistance in MECOM-amplified ovarian cancer. Cell Death Discov. (2025) 11:326. doi: 10.1038/s41420-025-02618-2, PMID: 40664642 PMC12264112

[101] Kiehlmeier S Rafiee MR Bakr A Mika J Kruse S Muller J . Identification of therapeutic targets of the hijacked super-enhancer complex in EVI1-rearranged leukemia. Leukemia. (2021) 35:3127–38. doi: 10.1038/s41375-021-01235-z, PMID: 33911178 PMC8550965

[102] Gulla S Sharma T Gardner E Li C Purohit TA Xue C . MECOM function is critical for AR-driven treatment-resistant prostate cancer. Cancer Res. (2026). doi: 10.1158/0008-5472.CAN-25-1720, PMID: 41529070 PMC13136877

[103] Ma Y Kang B Li S Xie G Bi J Li F . CRISPR-mediated MECOM depletion retards tumor growth by reducing cancer stem cell properties in lung squamous cell carcinoma. Mol Ther. (2022) 30:3341–57. doi: 10.1016/j.ymthe.2022.06.011, PMID: 35733338 PMC9637721

[104] Kataoka K Sato T Yoshimi A Goyama S Tsuruta T Kobayashi H . Evi1 is essential for hematopoietic stem cell self-renewal, and its expression marks hematopoietic cells with long-term multilineage repopulating activity. J Exp Med. (2011) 208:2403–16. doi: 10.1084/jem.20110447, PMID: 22084405 PMC3256960

[105] Lv J Meng S Gu Q Zheng R Gao X Kim JD . Epigenetic landscape reveals MECOM as an endothelial lineage regulator. Nat Commun. (2023) 14:2390. doi: 10.1038/s41467-023-38002-w, PMID: 37185814 PMC10130150

[106] Konantz M Andre MC Ebinger M Grauer M Wang H Grzywna S . EVI-1 modulates leukemogenic potential and apoptosis sensitivity in human acute lymphoblastic leukemia. Leukemia. (2013) 27:56–65. doi: 10.1038/leu.2012.211, PMID: 22828445

[107] Bard-Chapeau EA Jeyakani J Kok CH Muller J Chua BQ Gunaratne J . Ecotopic viral integration site 1 (EVI1) regulates multiple cellular processes important for cancer and is a synergistic partner for FOS protein in invasive tumors. Proc Natl Acad Sci U.S.A. (2012) 109:2168–73. doi: 10.1073/pnas.1119229109, PMID: 22308434 PMC3277513

[108] Lou M Zou L Zhang L Lu Y Chen J Zong B . MECOM and the PRDM gene family in uterine endometrial cancer: bioinformatics and experimental insights into pathogenesis and therapeutic potentials. Mol Med. (2024) 30:190. doi: 10.1186/s10020-024-00946-0, PMID: 39468462 PMC11514642

[109] Ma XH Gao MG Cheng RQ Qin YZ Duan WB Jiang H . The expression level of EVI1 and clinical features help to distinguish prognostic heterogeneity in the AML entity with EVI1 overexpression. Cancer Lett. (2025) 615:217547. doi: 10.1016/j.canlet.2025.217547, PMID: 39956382

[110] Voit RA Tao L Yu F Cato LD Cohen B Fleming TJ . A genetic disorder reveals a hematopoietic stem cell regulatory network co-opted in leukemia. Nat Immunol. (2023) 24:69–83. doi: 10.1038/s41590-022-01370-4, PMID: 36522544 PMC9810535

[111] Fukuda S Hoggatt J Singh P Abe M Speth JM Hu P . Survivin modulates genes with divergent molecular functions and regulates proliferation of hematopoietic stem cells through Evi-1. Leukemia. (2015) 29:433–40. doi: 10.1038/leu.2014.183, PMID: 24903482 PMC4258188

[112] Chen P Price C Li Z Li Y Cao D Wiley A . miR-9 is an essential oncogenic microRNA specifically overexpressed in mixed lineage leukemia-rearranged leukemia. Proc Natl Acad Sci U.S.A. (2013) 110:11511–6. doi: 10.1073/pnas.1310144110, PMID: 23798388 PMC3710804

[113] Mittal N Li L Sheng Y Hu C Li F Zhu T . A critical role of epigenetic inactivation of miR-9 in EVI1(high) pediatric AML. Mol Cancer. (2019) 18:30. doi: 10.1186/s12943-019-0952-z, PMID: 30813931 PMC6391809

[114] Christodoulou C Spencer JA Yeh SA Turcotte R Kokkaliaris KD Panero R . Live-animal imaging of native haematopoietic stem and progenitor cells. Nature. (2020) 578:278–83. doi: 10.1038/s41586-020-1971-z, PMID: 32025033 PMC7021587

[115] Holmfeldt P Pardieck J Saulsberry AC Nandakumar SK Finkelstein D Gray JT . Nfix is a novel regulator of murine hematopoietic stem and progenitor cell survival. Blood. (2013) 122:2987–96. doi: 10.1182/blood-2013-04-493973, PMID: 24041575 PMC3811173

